# Sex‐ and Male‐Morph‐Specific Variation in Brain Mass and Cell Number Scaling in Solitary *Centris pallida* (Hymenoptera: Apidae) Bees

**DOI:** 10.1002/cne.70163

**Published:** 2026-04-17

**Authors:** Meghan Barrett, R. Keating Godfrey

**Affiliations:** ^1^ Department of Biology Indiana University Indianapolis Indianapolis Indiana USA; ^2^ Neuroscience Program Middlebury College Middlebury Vermont USA

**Keywords:** alternative reproductive tactics, brain mass, cell densities, isotropic fractionation, optic lobes, sex differences, solitary bees

## Abstract

Intraspecific variation in behavior is associated with variable brain resource allocation patterns: There is frequently increased tissue investment in discrete regions that support fitness‐relevant cognitive abilities. However, the relationships between tissue volume and actual cell numbers have rarely been explored for insects due to methodological hurdles recently addressed via the application of isotropic fractionation. In solitary desert *Centris pallida* (Hymenoptera: Apidae) bees, there are two major levels of intraspecific variation: sex (males vs. females) and male morph (as a result of alternative reproductive tactics, large morph and small morph males rely on scent or sight, respectively, for mate location). Using isotropic fractionation, we separately analyzed optic lobe (OL) and central brain (CB) cell numbers of males and females to determine the impacts of sex and morph on brain cell allometry. Female bees’ brains were bigger and had higher cell numbers and cell densities than males of the same size. In both sexes, total brain cell number increased with brain size, driven by increases in OL cell numbers. Between male morphs, we found that OL masses were relatively larger in small‐morph males, consistent with the relationship between body size and OL volumes reported in prior studies. However, small‐morph *C. pallida* males had fewer total cells (as represented by cell nuclei) and reduced cell density, in their OLs. Together, these data suggest that there is intraspecific and brain‐region‐specific variation in brain cell numbers and that variation in brain tissue volume may not match other levels of neural organization like brain cell numbers/densities.

## Graphical abstract

We counted the number of cells in bees’ brains. Female *C. pallida* bees have more neurons in their brains overall than males. Small male morph bees had fewer cells and reduced densities in the visual input brain regions than large morph males, despite specializing on sight to find mates.

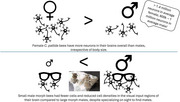

## 1 Introduction

Neural tissue is metabolically costly to produce and maintain; neuroecology theory thus predicts that energy limitation will cause investment in functionally discrete brain regions to be correlated with the cognitive and sensory demands imposed by an organism's behavior or environment (Aiello and Wheeler 1995; Sherry 2006; Niven and Laughlin 2008; Liao et al. 2016; Niven 2016; Luo et al. 2017). Variation in environment or behavior can drive differences in brain resource allocation patterns across or within species, with increased tissue investment in regions that support fitness‐relevant cognitive abilities (Healy and Guilford 1990; Warrant and Locket 2004; Soares and Niemiller 2013; Rehan et al. 2015; Montgomery and Ott 2015; Bulova et al. 2016; Stöckl et al. 2016; O'Donnell et al. 2017, 2018, 2021; Tierney et al. 2017; Keesey et al. 2019; Sheehan et al. 2019; Özer and Carle 2020; Baudier et al. 2022).

Many studies have demonstrated the relationship between relative brain volume or mass and a variety of cognitive demands; however, the relationship between tissue volume and actual cell numbers (e.g., processing power) has rarely been explored for insects due to methodological hurdles recently addressed via the application of isotropic fractionation (Herculano‐Houzel and Lent 2005) to insect brains (Godfrey et al. 2021). Cell counts are an important addition to our understanding of how brain investment relates to behavioral ability and energy limitation—in primates, the number of neurons better correlates with advanced behaviors and brain energy consumption than brain size (Herculano‐Houzel 2011). Cell populations are determined by the number and division cycles of neural precursor cells; these cells divide a specific number of times within each brain region of a species, generating species‐specific regional and total brain cell numbers (Urbach and Technu 2003).

Where documented, insect brain cell numbers are characterized as species‐specific averages, and little is known about intraspecific variation, particularly as it relates to size scaling or behavior. For example, in size‐polymorphic bumble bees (*Bombus* spp.), body size correlates with division of colony tasks (Spaethe and Weidenmüller 2002; Goulson 2010) and brain volume scales with body size (Mares et al. 2005). Although the size polymorphism in social bees is established via differences in larval provisioning and rearing environment (Chole et al. 2019), whether changes in brain size are due to scaling of cell number, cell size, or some combination has not been determined. In line with the stereotyped development described for many insects, such species could show stable cell numbers in different brain regions irrespective of behavioral or body size variation. In this case, brain volumetric or mass differences might be due to cell growth during development or neural plasticity in adulthood (Riveros and Gronenberg 2010). Conversely, neural precursor cells could divide variable numbers of times, or cells could be culled during development, leading to differences in cell numbers across individuals or in specific brain regions, potentially resulting in differences in function and behavioral abilities.

A recent study in black soldier flies (*Hermetia illucens*, Diptera: Stratiomyidae; BSF) found that neither optic lobe (OL) nor central brain (CB) cell numbers varied with body size in males or females, supporting the idea that cell number is fixed irrespective of body size within a single sex and species (Barrett et al. 2022). However, body mass is not especially variable amongst BSF of a single sex and population (less than twofold variation for both sexes in Barrett et al. 2022), and more polymorphic insect species have yet to be tested intraspecifically. By contrast, categorical behavioral variation, such as in visual mate location behaviors between male and female BSF and drone and queen western honey bees, was associated with increases in OL, but not CB, cell numbers in males (Witthöft 1967, Barrett et al. 2022). Thus, the limited data currently available on insect brain cell numbers suggest both categorical behavioral variation and allometric scaling should be given further consideration in predicting how brain cell numbers may vary intraspecifically.

*Centris pallida* (Hymenoptera: Apidae) bees show sex‐specific body size allometries and have two distinct male morphs with unique physical characteristics and mating strategies, making them an apt system to better understand the roles of body size and behavioral variation in brain cell number scaling. Both males and females have significant variation in body size; prior research showed males varied from at least 0.11 to 0.35 g in mass (Barrett et al. 2021), and females from at least 0.14 to 0.28 g (Barrett et al. 2023). Male *C. pallida* are strongly dimorphic, using alternative reproductive tactics with variable sensory mate location behaviors that have been shown to correlate with relative investment patterns in the brain's peripheral sensory processing regions (Barrett et al. 2021). These behavioral categories are size‐associated: Large‐morph males rely on a chemosensory mate location strategy to find females emerging from underground natal nests, whereas small‐morph males primarily use a visual mate location strategy to find females flying in the sky (Alcock 1976, 1984, 1997; Alcock et al. 1976, 1977; Houston 1991; Simmons et al. 2003). Large‐morph males have increased relative antennal lobe volumes (which receive chemosensory information from the antennae), whereas small‐morph males have relatively larger OL volumes (which receive visual information from the eyes; Barrett et al. 2021). Further, small morph males have absolutely smaller, but relatively larger, brain volumes compared to large‐morph males (Barrett 2022), demonstrating patterns consistent with Haller's Rule (Rensch 1948).

Here, we tested whether males and females differ in brain mass or the relative size of OL and CB regions. We looked at the effects of body size on brain allometry between the sexes and assessed whether differences in brain cell number are driven by region‐specific densities. Finally, we moved beyond sex‐specific differences in brain investment to test whether male dimorphism generated further specialization at the cellular level among males, beyond the volumetric shifts characterized by Barrett et al. (2021). Because this system features a behavioral dimorphism that leverages a morphological polymorphism, we were able to assess differences in the scaling of brain region masses and cell numbers/densities in relation to body size, as well as the effect of categorical variation in behavior on cell numbers. We hypothesized that brain cell numbers and densities are not fixed in behaviorally polymorphic species. We therefore predicted there would be differences in cell number and density between the male morphs. We also predicted we would see differences in cell numbers and densities between males and females (as seen in other species).

## 2 Materials and Methods

### 2.1 Specimen Collection and Body Mass

We collected specimens in April 2020 and 2022 at Tumamoc Hill in Tucson, Arizona, near Silvercroft Wash (N32.2232, W111.0091), where aggregations of this species have been studied since 2016. We briefly chilled bees on ice before cutting the head from the thorax with a razor; heads were placed immediately into Prefer fixative (Anatech Ltd.), which we refrigerated until dissection. We classified *C. pallida* male morphs as in Barrett et al. (2021). Briefly, “large” morph males (sometimes called metanders) were found patrolling, digging, or fighting and had the grey/white coloration and bulging hind leg morphology distinct to this morph; “small” morph males (sometimes called hoverers) were found hovering near vegetation or sometimes patrolling, but they did not have the distinct coloration or morphology of the large morph males (Alcock 1976; Alcock et al. 1977; Snelling 1984). Categorical morph assignment in this species is largely based on behavioral strategy and coloration, as was done in our study.

For some samples, we were able to obtain wet body mass directly; however, due to COVID‐19‐related laboratory access restrictions in April 2020, we could not obtain wet mass for all individuals and had to estimate the mass by using a dry‐to‐wet mass conversion (determined by taking a set of specimens from the same population with known wet mass and drying them for 3 days in a convection oven at 60°C; Figure S1; [wet weight] = 3.06 [headless dry weight] + 0.02; *F* = 5645, df = 85, *R* ^2^ = 0.99, *p* < 0.0001).

### 2.2 Dissection and Isotropic Fractionation

We dissected brains in phosphate buffer saline (PBS; MP Biomedicals LLC). We separated OLs from the CB and removed the retinas. We stored dissected brains at 4°C in PBS for 24 h. We then blotted brains dry carefully with a Kimwipe and weighed the OLs and CB separately to the nearest 0.01 mg on a Mettler Toledo AT261 (Marshall Scientific, Hampton, NH, USA) analytical balance in 80% glycerol to minimize evaporation. If an OL was lost or damaged during dissection (*n* = 2 females), we doubled the mass of the remaining OL.

As in Godfrey et al. (2021), we homogenized brain tissue using a glass tissue homogenizer with sodium citrate and Triton X detergent solution, diluted with PBS. We labeled nuclei with the fluorescent nucleic acid probe, SYTOX Green (Thermo Fischer Scientific), then counted them with a hemocytometer under epifluorescence using a 40×/0.65 M27 objective on a Zeiss Axioplan microscope. We counted 12 subsamples of each homogenized brain region and averaged to obtain a mean number of cells in that brain region. We obtained cell density by dividing the mean number of nuclei for a given brain region by the mass of that brain region (assuming one nucleus per brain cell). We counted each adult's CB and pooled OLs (*n* = 9 small‐morph males, *n* = 10 large‐morph males; *n* = 12 females for OL, 14 for CB, due to loss of an OL during dissection in two cases).

### 2.3 Data Analysis

We used GraphPad Prism v. 9.1.2 (GraphPad Prism for Windows 2021; RRID: SCR_002798) for all statistical analyses. We used Grubbs test (*α* = 0.01) and ROUT method (*Q* = 5%) analyses to identify outliers in the dataset; the same outlier male individual was identified using both methods (likely a result of a dilution error, given the individual's normal brain mass and abnormal cell densities/counts); we excluded this individual from all brain cell (but not brain mass and body size) analyses. However, we plotted the outlier in grey on all graphs, and statistical analyses including this individual can be found in Table S1.

As a result of the significant variation in body size in both males and females, we analyze for sex‐based differences by comparing their scaling relationships and not their means. By contrast, given that there is categorical variation in male morphs, we do analyze differences in mean characteristics between morphs.

We used Shapiro–Wilk normality tests and an *F*‐test for equal variance to determine if data met the assumptions of parametric tests. We used unpaired *t*‐tests to analyze categorical differences between male morphs in head width, total and relative brain mass, relative OL:CB mass, total brain cell numbers, and relative OL:CB cell number. Body mass data were not normally distributed, so we used a Mann–Whitney *U* nonparametric test. We used a Brown–Forsythe ANOVA with a Dunnett's T3 MCT (reporting multiplicity adjusted *p* values) to analyze categorical differences between male morphs in OL and CB mass, and OL and CB cell numbers (due to unequal variance), whereas a one‐way ANOVA with Bonferroni MCT was used to analyze OL and CB cell density.

Linear regressions were used to analyze the relationship between wet weight and headless dry weight; log (body mass) and log (brain region mass); brain mass and cell numbers; and brain mass and cell densities. Extra sum‐of‐square *F*‐tests were used for all comparisons of fit.

## 3 Results

### 3.1 Body Mass and Head Width

Large‐morph males had greater body mass than small‐morph males (Mann–Whitney test; *U* = 0, *p* < 0.0001). Large males ranged in body mass from 0.24 to 0.32 g (mean = 0.27 ± 0.03 g); small males ranged in body mass from 0.12 to 0.15 g (mean = 0.14 ± 0.01 g). Females ranged in body mass from 0.14 to 0.30 g (mean = 0.22 ± 0.06 g).

Large‐morph males had wider heads than small‐morph males (unpaired *t*‐test; *t* = 11.08, df = 16, *p* < 0.0001). Large males ranged in head width from 5.37 to 5.89 mm (mean = 5.58 ± 0.17 mm); small males ranged in head width from 4.57 to 4.98 mm (mean = 4.77 ± 0.15 mm). Females’ heads were 4.74–5.94 mm wide (mean = 5.34 ± 0.40 mm).

### 3.2 Brain Mass Scaling Between Sexes

Mean and ranges of female and male morph brain masses are reported in Table 1.

**TABLE 1:** Mean ± *SD* (and ranges) of male and female brain masses (mg).

	Total brain	OL	CB
Females	4.07 ± 0.54 (3.27–4.87)	2.53 ± 0.40 (1.96–3.18)	1.54 ± 0.17 (1.26–1.82)
Males (all)	3.63 ± 0.31 (3.12–4.13)	2.49 ± 0.19 (2.13–2.78)	1.14 ± 0.13 (0.97–1.35)
Large males	3.85 ± 0.19 (3.55–4.13)	2.61 ± 0.12 (2.41–2.78)	1.24 ± 0.08 (1.14–1.35)
Small males	3.40 ± 0.23 (3.12–3.86)	2.37 ± 0.17 (2.13–2.68)	1.04 ± 0.06 (0.97–1.18)

Abbreviations: CB, central brain; OL, optic lobe.

**FIGURE 1:**
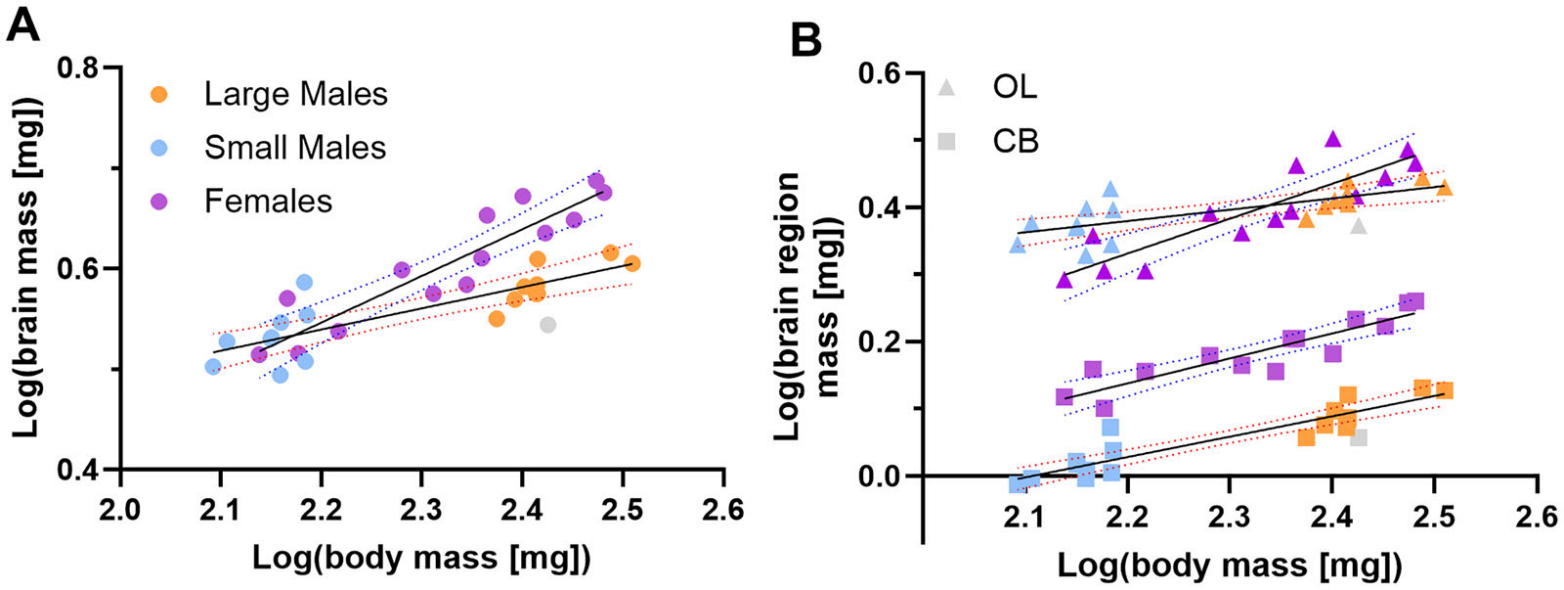
Brain mass scales differently with body size in male and female *Centris pallida*, and in OLs, the CB, and the brain overall. (A) The brain scaled hypoallometrically with body mass in both males and females; however, male brains grew more slowly as body mass increased. For males, solid black line with 95% CI dotted red lines (log(total brain mass) = 0.21 [log(body mass)] + 0.08 (*R* ^2^ = 0.68, *F* = 34.73, df = 16, *p* < 0.0001)). In females, solid black line with 95% CI dotted blue lines (log(total brain mass) = 0.46 [log(body mass)] − 0.48 (*R* ^2^ = 0.85, *F* = 69.49, df = 12, *p* < 0.0001)). (B) Females had larger CB masses compared to males of the same body size, represented by the increased elevation of the best fit line (*F* = 256.1, df = 29, *p* < 0.0001). However, there was no difference in scaling coefficient (*b *= 0.33, pooled; *F* = 1.35, df = 28, *p* = 0.25). However, female OL mass had an increased scaling coefficient compared to male OL mass (females, *b *= 0.52; males, *b *= 0.17; *F* = 18.33, *R* ^2^ = 0.53, *p* = 0.0006). Grey dot = ROUT‐identified male outlier in IF analyses, excluded from all statistics. CB, central brain; OL, optic lobe.

Large and small males did not differ in the scaling relationship between brain mass and body mass (extra sum‐of‐squares *F*‐test; TB: *F* = 0.8481, df = 14, *p* = 0.45; OL: *F* = 0.60, df = 14, *p* = 0.56; CB: *F* = 1.42, df = 14, *p* = 0.28) and were thus regressed together. Brain mass scaled hypoallometrically with body mass in both males and females; however, the scaling coefficients were significantly different between males and females (Figure 1A, *F*‐test; *F* = 15.26, df = 38, *p* = 0.0005), with the relationship of brain size to body size increasing less steeply in males than females. The scaling coefficient (*b*) describing the relationship between brain and body mass in males was 0.21 (log(total brain mass) = 0.21 [log(body mass)] + 0.08, *R* ^2^ = 0.68, *F* = 34.73, df = 16, *p* < 0.0001) and in females was 0.46 (log(total brain mass) = 0.46 [log(body mass)] − 0.48, *R* ^2^ = 0.85, *F* = 69.49, df = 12, *p* < 0.0001).

This difference in total brain scaling relationships between the sexes was driven by the OLs and not the CB (Figure 1B). Although females had larger CBs compared to males of the same body size (represented by the difference in elevation between the lines of best fit for males and females, *F* = 256.1, df = 29, *p* < 0.0001), there was no difference in the scaling coefficients of male and female CBs (*b *= 0.33, pooled; *F* = 1.35, df = 28, *p* = 0.25). The line of best fit for males was log(CB mass) = 0.30 log(brain mass) − 0.64 (*R* ^2^ = 0.85, *F* = 89.55, df = 16, *p* < 0.0001). The line of best fit for females was log(CB mass) = 0.37 log(body mass) − 0.68 (*R* ^2^ = 0.81, *F* = 52.39, df = 12, *p* < 0.0001).

Conversely, females had a substantially increased scaling coefficient for their OLs (log(OL mass) = 0.52 log(body mass) − 0.68, *F* = 42.26, *R* ^2^ = 0.78, df = 12, *p* < 0.0001) compared to males (log(OL mass) = 0.17 [log(body mass)] + 0.008, *F* = 18.33, *R* ^2^ = 0.53, df = 16, *p* = 0.0006), though both were still hypoallometric. Interestingly, there was a “crossover” point in the lines of best fit for male and female OL masses, suggesting a likely interaction between size and sex; OL size is more tightly constrained at small body sizes for males than females.

### 3.3 Brain Mass Differences Between Male Morphs

**FIGURE 2:**
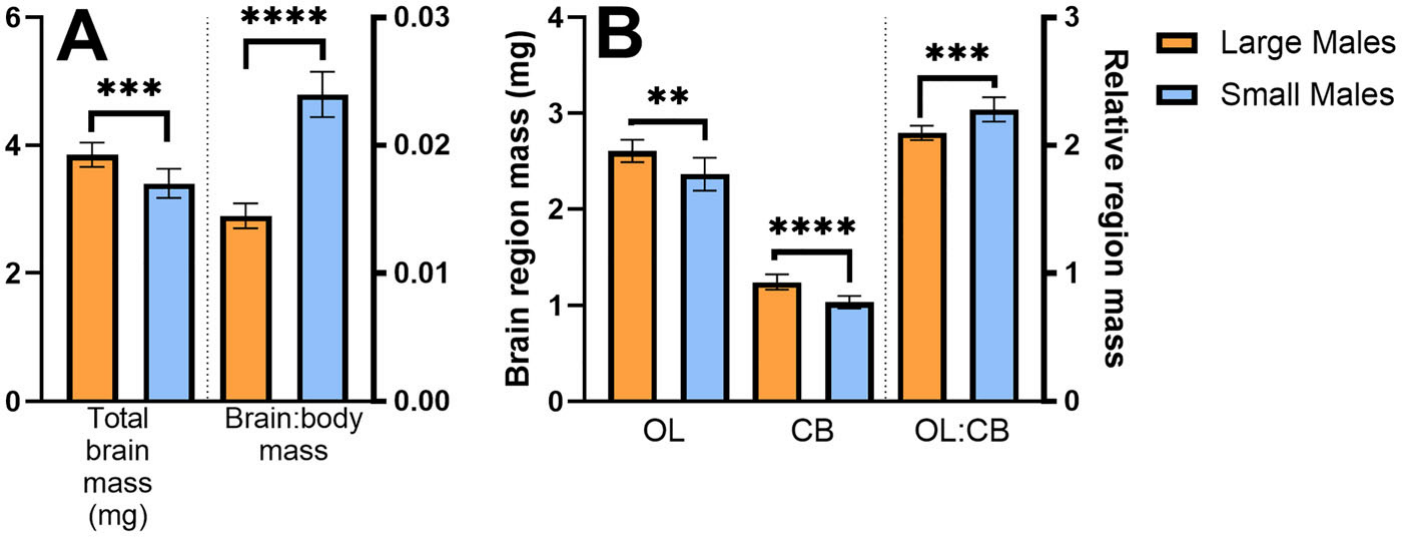
Total and relative brain region masses differ between male morphs in *Centris pallida*. (A) Large‐morph (orange) males have significantly larger total (*t* = 4.53, df = 16, *p* = 0.0003) but reduced relative (*t* = 14.10, df = 16, *p* < 0.0001) brain mass compared to small‐morph males (blue). (B) Large‐morph males (orange) had greater total OL (Brown–Forsythe ANOVA; *F* = 418.6, *p* < 0.0001; Dunnett's T3; *t* = 3.47, df = 14.13, *p* = 0.0074) and CB (*t* = 6.23, df = 15.61, *p* < 0.0001) masses than small‐morph males (blue). Relative OL mass (OL:CB) was larger in small males (*t* = 4.95, df = 16, *p* = 0.0001), due to differences in the allometric scaling coefficients of each brain region. Means with error bars representative of standard deviations. CB, central brain; OL, optic lobe. **p < 0.01; ***p < 0.001; ****p < 0.0001.

Large‐morph males had greater total, but less relative, brain mass compared to small‐morph males (Figure 2A: unpaired *t*‐tests; total brain mass: *t* = 4.53, df = 16, *p* = 0.0003; relative brain mass: *t* = 14.10, df = 16, *p* < 0.0001).

Total OL (Figure 2B: Brown–Forsythe ANOVA; *F* = 418.6, *p* < 0.0001; Dunnett's T3; *t* = 3.47, df = 14.13, *p* = 0.0074) and CB (*t* = 6.23, df = 15.61, *p* < 0.0001) mass was larger in large males, but relative OL mass (OL:CB) was larger in small males (*t* = 4.95, df = 15.61, *p* < 0.0001).

### 3.4 Brain Cell Numbers Between Sexes

Mean and ranges of female and male morph cell numbers and densities are reported in Table 2.

**TABLE 2:** Mean ± *SD* (and ranges) of male and female cell numbers and cell densities.

	Total brain	OLs	CB
**Cell numbers (in millions)**			
Females	1.44 ± 0.12 (1.24–1.58)	0.98 ± 0.09 (0.82–1.12)	0.46 ± 0.05 (0.41–0.57)
Males (all)	0.89 ± 0.12 (0.66–1.04)	0.66 ± 0.12 (0.42–0.82)	0.23 ± 0.01 (0.20–0.25)
Large males	0.98 ± 0.05 (0.91–1.04)	0.75 ± 0.05 (0.68–0.82)	0.22 ± 0.01 (0.20–0.24)
Small males	0.80 ± 0.09 (0.66–0.93)	0.57 ± 0.09 (0.42–0.71)	0.23 ± 0.02 (0.20–0.25)
**Cell densities (in cells/µg)**			
Females	359.8 ± 31.4 (328.4–437.5)	394.2 ± 38 (336–457)	302.6 ± 36.7 (260.8–406.3)
Males (all)	244 ± 20.8 (207.2–276.5)	264.6 ± 35.6 (191.5–319.3)	200.2 ± 25.1 (151.8–242.9)
Large males	253.8 ± 17.4 (227.9–276.5)	289 ± 20.7 (264.9–319.3)	180.1 ± 17.1 (151.8–200.5)
Small males	234.3 ± 20.2 (207.2–264.3)	240.3 ± 30.6 (191.5–285.4)	220.3 ± 11.8 (205–242.9)

Abbreviations: CB, central brain; OL, optic lobe.

**FIGURE 3:**
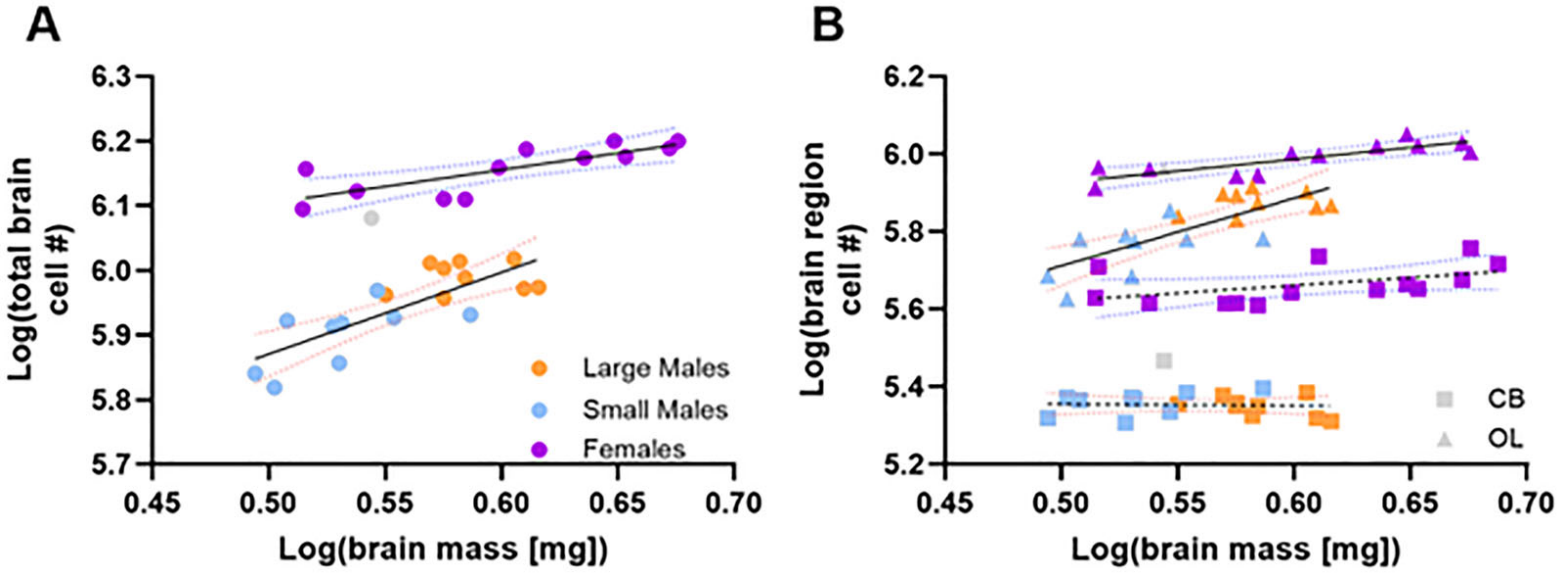
Brain cell numbers scale differently with brain size in male and female *Centris pallida*, and in OLs, the CB, and the brain overall. (A) Total brain cell numbers scaled hypoallometrically with brain mass in females and hyperallometrically in males (*F* = 7.29, df = 26, *p* = 0.012). For males, solid black line with 95% CI dotted red lines (log(total brain cell #) = 1.26 [log(brain mass)] + 5.24; *R* ^2^ = 0.62, *F* = 26.66, df = 16, *p* < 0.0001). In females, solid black line with 95% CI dotted blue lines (log(total brain cell #) = 0.52 ([log(brain mass)] + 5.85; *R* ^2^ = 0.62, *F* = 16.49, df = 10, *p* = 0.0023). (B) CB cell numbers did not change with brain mass in either males (Figure 4B: *F* = 0.04, df = 16, *p* = 0.84) or females (*F* = 3.62, df = 12, *p* = 0.08), though females had a greater number of cells than males (*F* = 409.1, df = 29, *p* < 0.0001). Female OL cell numbers scaled hypoallometrically with brain mass, and male OL cell numbers scaled hyperallometrically (*F* = 9.32, df = 26, *p* = 0.0052). In males, log(OL cell #) = 1.74 [log(brain mass)] + 4.84 (*R* ^2^ = 0.61, *F* = 24.85, df = 16, *p* = 0.0001); in females, log(OL cell #) = 0.61 [log(brain mass)] + 5.62 (*R* ^2^ = 0.70, *F* = 23.36, df = 10, *p* = 0.0007). Grey dot = ROUT‐identified male outlier in IF analyses, excluded from all statistics. CB, central brain; OL, optic lobe.

Large and small males did not differ in the scaling relationship between brain mass and brain cell number (comparison of fits test; TB: *F* = 3.17, df = 14, *p* = 0.07; OL: *F* = 2.71, df = 14, *p* = 0.10; CB: *F* = 2.30, df = 14, *p* = 0.14) and were thus regressed together. Total brain cell number scaled hypoallometrically with brain mass in females (Figure 3A: log(total brain cell #) = 0.52 ([log(brain mass)] + 5.85; *R* ^2^ = 0.62, *F* = 16.49, df = 10, *p* = 0.0023) but hyperallometrically in males (log(total brain cell #) = 1.26 [log(brain mass)] + 5.24; *R* ^2^ = 0.62, *F* = 26.66, df = 16, *p* < 0.0001); males brain cell numbers increased faster with brain size than females (*F* = 7.29, df = 26, *p* = 0.012); however, females had more total brain cells than males of the same brain mass. CB cell numbers did not change with brain mass in either males (Figure 3B: *F* = 0.04, df = 16, *p* = 0.84) or females (*F* = 3.62, df = 12, *p* = 0.08), though females had a greater number of cells than males (*F* = 409.1, df = 29, *p* < 0.0001). Thus, differences in the scaling of total brain cell numbers between the sexes were driven by the OLs where females had hypoallometric scaling (log(OL cell #) = 0.61 [log(brain mass)] + 5.62; *R* ^2^ = 0.70, *F* = 23.36, df = 10, *p* = 0.0007) and males had hyperallometric scaling (log(OL cell #) = 1.74 [log(brain mass)] + 4.84; *R* ^2^ = 0.61, *F* = 24.85, df = 16, *p* = 0.0001); male OL cell numbers increased faster with brain size than females (*F* = 9.32, df = 26, *p* = 0.0052) even though females had more OL cells at the same brain mass (Figure 3B).

### 3.5 Brain Cell Densities Between Sexes

Large and small males differed in the scaling relationship between brain mass and CB density (*F* = 8.16, df = 14, *p* = 0.0045), with a nonsignificant trend towards difference in OL cell density (*F* = 3.52, df = 14, *p* = 0.058) and total brain cell density (*F* = 3.17, df = 14, *p* = 0.073). As a result, we did not regress large and small males’ cell densities together to compare to females.

**FIGURE 4:**
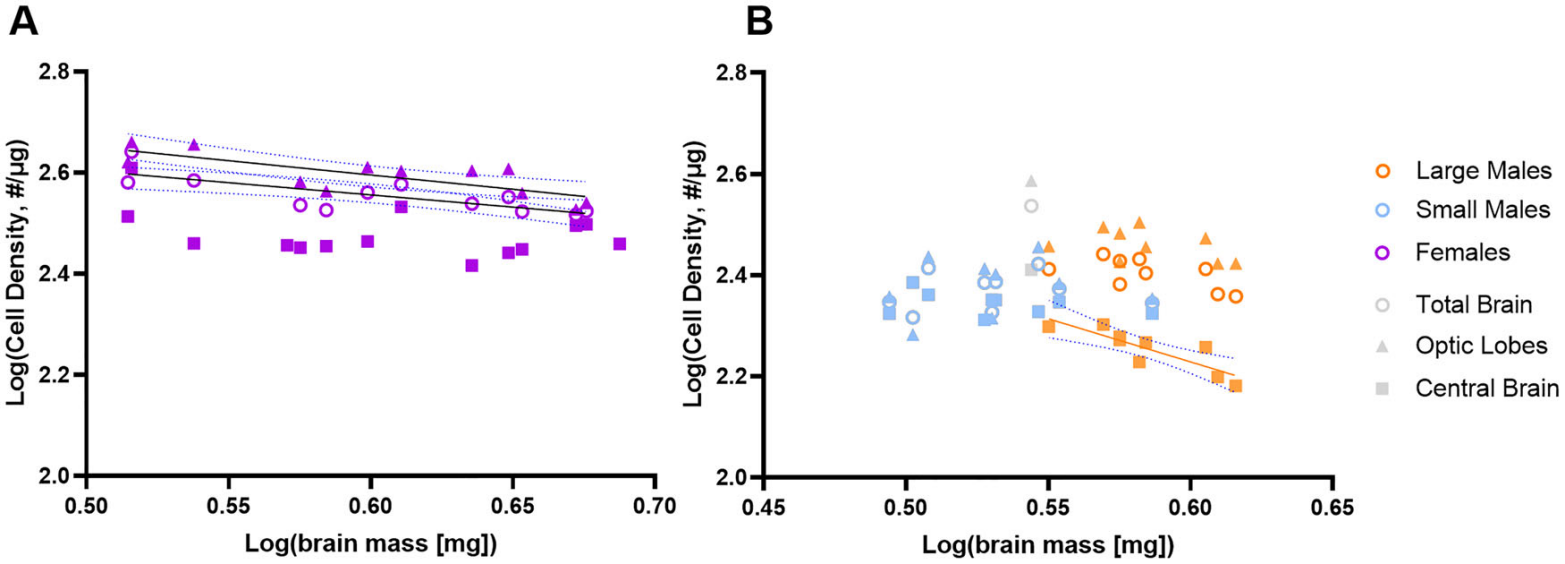
Brain cell densities scale differently between males and females and across brain regions. (A) Female total brain cell density (log(TB cell density) = −0.48 [log(brain mass)] + 2.85; *R* ^2^ = 0.59, *F* = 14.44, df = 10, *p* = 0.0035) and OL cell density (log(OL cell density) = −0.56 [log(brain mass)] + 2.93; *R* ^2^ = 0.61, *F* = 15.35, df = 10, *p* = 0.0029) decreased with brain mass; CB cell density did not change with brain mass in females (*F* = 2.43, df = 12, *p* = 0.15). (B) Neither large‐morph nor small‐morph male total brain nor OL cell density changes with brain mass (all *p* > 0.067). However, CB cell density decreased with brain mass in large‐morph males (log(CB cell density) = −1.70 [log(brain mass)] + 3.25; *R* ^2^ = 0.73, *F* = 19.31, df = 7, *p* = 0.0032). Grey dot = ROUT‐identified male outlier in IF analyses, excluded from all statistics. Significant trendlines are shown as black (female) or orange (large‐morph male) solid lines, with 95% CI as blue dotted lines.

Females had more dense brains overall and in each brain region than males of the same brain mass. There was a negative relationship between brain mass and total brain cell density in females (Figure 4; log(TB cell density) = −0.48 [log(brain mass)] + 2.85, *F* = 14.44, df = 10, *p* = 0.0035), but there was no such relationship in large‐morph males (*F* = 4.69, df = 7, *p* = 0.067) or small‐morph males (*F* = 0.07, df = 7, *p* = 0.80). Similarly, there was a negative relationship between brain mass and OL cell density in females (log(OL cell density) = −0.56 [log(brain mass)] + 2.93; *R* ^2^ = 0.61, *F* = 15.35, df = 10, *p* = 0.0029), but no relationship between brain mass and OL cell density in large‐morph males (*F* = 1.51, df = 7, *p* = 0.26) or small‐morph males (*F* = 0.18, df = 7, *p* = 0.68). Conversely, there was no relationship between brain mass and CB cell density in females (*F* = 2.43, df = 12, *p* = 0.15) or small‐morph males (*F* = 1.19, df = 7, *p* = 0.31); however, there was a negative relationship between brain mass and CB cell density in large‐morph males (log(CB cell density) = −1.70 [log(brain mass)] + 3.25; *R* ^2^ = 0.73, *F* = 19.31, df = 7, *p* = 0.0032).

### 3.6 Brain Cell Numbers and Densities Between Male Morphs

**FIGURE 5:**
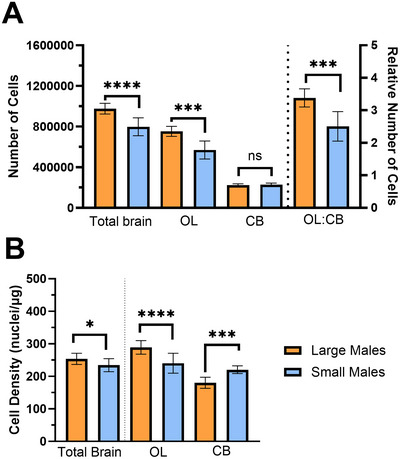
OL and CB cell numbers, and cell densities, in male *Centris pallida* bees. (A) Large‐morph males (orange) had more cells in their whole brain (unpaired *t*‐test; *t* = 5.20, df = 16, *p* < 0.0001) than small‐morph males (blue), driven by increases in the number of OL cells (Brown–Forsythe ANOVA; *F* = 231.2, *p* < 0.0001; Dunnett's T3; *t* = 5.41, df = 12.49, *p* = 0.0003) as there was no difference in the number of cells in the CB between morphs (*t* = 0.79, df = 15.47, *p* = 0.68). Large‐morph males had increased cell numbers in their OLs relative to their CB (*t* = 4.92, *p* = 0.0002). (B) Large‐morph males had denser OLs (ANOVA; *F* = 41.06, *p* < 0.0001; Bonferroni; *t* = 4.88, df = 32, *p* < 0.0001), but less dense CB (*t* = 4.03, *p* = 0.0006), than small‐morph males, resulting in a slightly denser brain overall (unpaired *t*‐test; *t* = 2.19, df = 16, *p* = 0.0434). Means with error bars representative of standard deviations. CB, central brain; ns, not significant; OL, optic lobe. *p < 0.05; ***p < 0.001; ****p < 0.0001.

Large‐morph males had more cells in their brains than small‐morph males (Figure 5A: unpaired *t*‐test; *t* = 5.20, df = 16, *p* < 0.0001); however, this difference was driven only by an increase in OL cell numbers (Brown–Forsythe ANOVA; *F* = 231.2, *p* < 0.0001; Dunnett's T3; *t* = 5.41, df = 12.49, *p* = 0.0003) and not CB cell numbers (*t* = 0.79, df = 15.47, *p* = 0.68). Large‐morph males thus had increased cell numbers in their OLs relative to their CB (*t* = 4.92, *p* = 0.0002), as OL cell numbers increased hyperallometrically with brain mass, but CB numbers did not change (Figure 3B; *F* = 0.04, *R* ^2^ = 0.003, *p* = 0.84). Large‐morph males had increased OL cell density (Figure 5B: ANOVA; *F* = 41.06, *p* < 0.0001; Bonferroni; *t* = 4.88, df = 32, *p* < 0.0001) but decreased CB cell density (*t* = 4.03, *p* = 0.0006), resulting in an overall increase in cell density for large males in the brain overall (*t* = 2.19, df = 16, *p* = 0.0434).

## 4 Discussion

We found strong intraspecific differences in brain mass and cell numbers between sexes and male morphs of *C. pallida* bees, with the OLs playing a large role in generating many of these differences. Although brain mass scaled hypoallometrically with body mass in both sexes, female brains scaled more steeply than male brains (*b *= 0.21 males, *b *= 0.46 females); this was driven by OL scaling and not CB scaling (the scaling relationship was the same between the sexes; *b *= 0.33), though females had larger CBs compared to males of the same body mass. OL mass was ∼2/3 of brain mass (∼62% in females, ∼69% in males), similar to the white‐lined sphinx moth (Askamit et al. 2024) and black soldier fly (Barrett et al. 2022), but much higher than in many flies or nocturnal moths (Stöckl et al. 2016; Özer and Carle 2020). Although there are differences in OL mass scaling between the sexes (where small females have smaller OLs than small males, but large females have larger OLs than large males), females of all sizes had a larger CB mass than size‐comparable males.

Sex‐specific patterns were more complex when considering brain cell number: Females had much higher total brain cell numbers than males of the same mass (mean females: 1.44 million, males: 0.89 million), but total brain cell number scaled hypoallometrically in females (*b *= 0.52) and hyperallometrically in males (*b *= 1.26). This difference in scaling relationship was again entirely driven by the OLs, as CB cell numbers did not change with brain mass in either males or females, though females continued to have more cells in both their CB and OLs compared to males of the same size. It is possible that female *C. pallida* may have larger task repertoire sizes than males (mating as well as foraging, nesting, defense against nest parasites, etc.), which has been hypothesized to drive increased investment in higher order regions of the brain (O'Donnell et al. 2018, but see Baudier et al. 2022). However, much greater research on both the sex differences in task repertoire and the location of the larger number of cells in female CB compared to the males would be needed to support this hypothesis, and it may not explain the need for increased OL cells in females.

Interestingly, OL mass and cell scaling differences between males and females are unlikely to be driven by eye size or ommatidia number. Male *C. pallida* bees always have larger eyes than females of their size (and female eye size does not scale with body size, as does their OL mass and cell number). Further, absolute ommatidia number is greater in male *C. pallida* bees compared to females of their size, with males increasing both ommatidia number and largest facet size as they grew in body size (Barrett and O'Donnell 2026). Estimates of visual acuity based on external morphology suggest females have the same vertical and horizontal visual acuity in their frontodorsal hotspot as large males but improved visual acuity compared to small males (Barrett and O'Donnell 2026). How this visual acuity may be further altered by variation in OL cell density is currently unknown but worth further exploration. Altogether, these data suggest scaling relationships between multiple parts of the sensory system can be decoupled: eye size, ommatidia diameter and number, OL cell density/number, and OL volume do not always correlate. This flexibility across sensory system components is likely a key but underexplored driver of variation in sensory performance across insects.

Brain cell numbers for female *C. pallida* were comparable to other female Hymenoptera of similar body size (Godfrey et al. 2021), whereas males had dramatically lower total cell numbers than females (opposite to the pattern observed in honey bees, though male honey bee total cell numbers were largely driven by the OLs; Witthöft 1967). OL cells in *C. pallida* comprised 68% (females) or 74% (males) of total brain cells, comparable to the sphinx moth (65%; Askamit et al. 2024) and fruit flies (68%; Mu et al. 2022) and much lower than black soldier flies (∼88%; Barrett et al. 2022). Cell density is thus not homogenous among brain regions, or between sexes, in *C. pallida*. Our data are in line with data from black soldier flies, where OL cell numbers were the major driver of sex differences and CB cell numbers were fixed (Barrett et al. 2022). This may suggest that neural precursor cells responsible for producing brain cells in different areas have different developmental flexibility: cells that create CB regions may have less flexibility in the number of times they divide or number of cells they create than those in the OLs. Further research is necessary to explain how variable OL cell numbers are created within a single species during development and to explore why these differences occur in some species (e.g., BSF, *C. pallida*) and not others (e.g., no differences in the OLs between sexes of *Drosophila melanogaster*, many mosquitoes; Raji and Potter 2021; or sphinx moths, Askamit et al. 2024). Importantly, small changes in the cell numbers of different discrete neuropils within the CB regions (such as the mushroom bodies or central complex) would likely go undetected with our methods, despite potentially having measurable functional consequences, representing a technical limitation of the IF technique.

Differences were also observed between the male morphs in brain mass, matching prior research on volumetric differences: just as small male brains were found to have absolutely smaller but relatively larger OLs by volume (Barrett et al. 2021), so too were they found to have absolutely smaller but relatively larger OLs by mass. These data further corroborate the link between visual mate location strategies and increased investment in peripheral visual input tissue allocation in the species (Barrett et al. 2021). Large‐morph males had more cells in their brain, but this was only driven by an increase in OL cell numbers (with no difference in CB cell numbers as body size increased or between morphs). This resulted in higher OL cell density, and lower CB cell density, for large morph males compared to small morph males.

Altogether, our data provide further evidence outside honey bees that male hymenoptera have different brain size scaling relationships from female hymenoptera (within, and potentially across, species), with differences in the scaling of both region size and cell densities largely driven by differences in the OLs. However, there are also absolute differences in size and cell densities between the sexes that may be a result of both the CB and OLs. We show that intraspecific differences in fitness‐relevant sensory abilities, such as those underlying alternative reproductive tactic systems, can drive differences in brain mass and cell allometry in insects. Future research in this study system could reveal if nervous systems adapt intraspecifically to fine‐tune dimensions like transmission speed, noise, and energy consumption by changing cell size and number, with ultimate consequences for animal behavior and sensory ability. In addition, greater research into the development of insect nervous systems is necessary to elucidate the mechanisms underlying the intraspecific variability in OL cell number observed in numerous taxa (both between sexes and within a sex), given that variation in CB cell number is far less frequently detected.

## Funding

This work was supported by a National Science Foundation PRFB granted to MB (2109399); all findings and opinions are the authors’ and not reflective of the NSF.

## Conflicts of Interest

The authors declare no conflicts of interest.

## Supporting information




**Supplementary Information**: cne70163‐sup‐0001‐SuppMat.docx

**Supplementary Information**: cne70163‐sup‐0002‐SuppMat.xlsx

## Data Availability

Raw data are available as Supporting Information File 2.
