# Supplementary material for: Sex‐ and Male‐Morph‐Specific Variation in Brain Mass and Cell Number Scaling in Solitary Centris pallida (Hymenoptera: Apidae) Bees
Source: J Comp Neurol. 2026 Apr 17;534:e70163. doi: 10.1002/cne.70163 (PMC13088941; doi:10.1002/cne.70163)
Supplement: Supplementary file 1 — Supplementary Information: cne70163‐sup‐0001‐SuppMat.docx [file CNE-534-e70163-s001.docx]

**
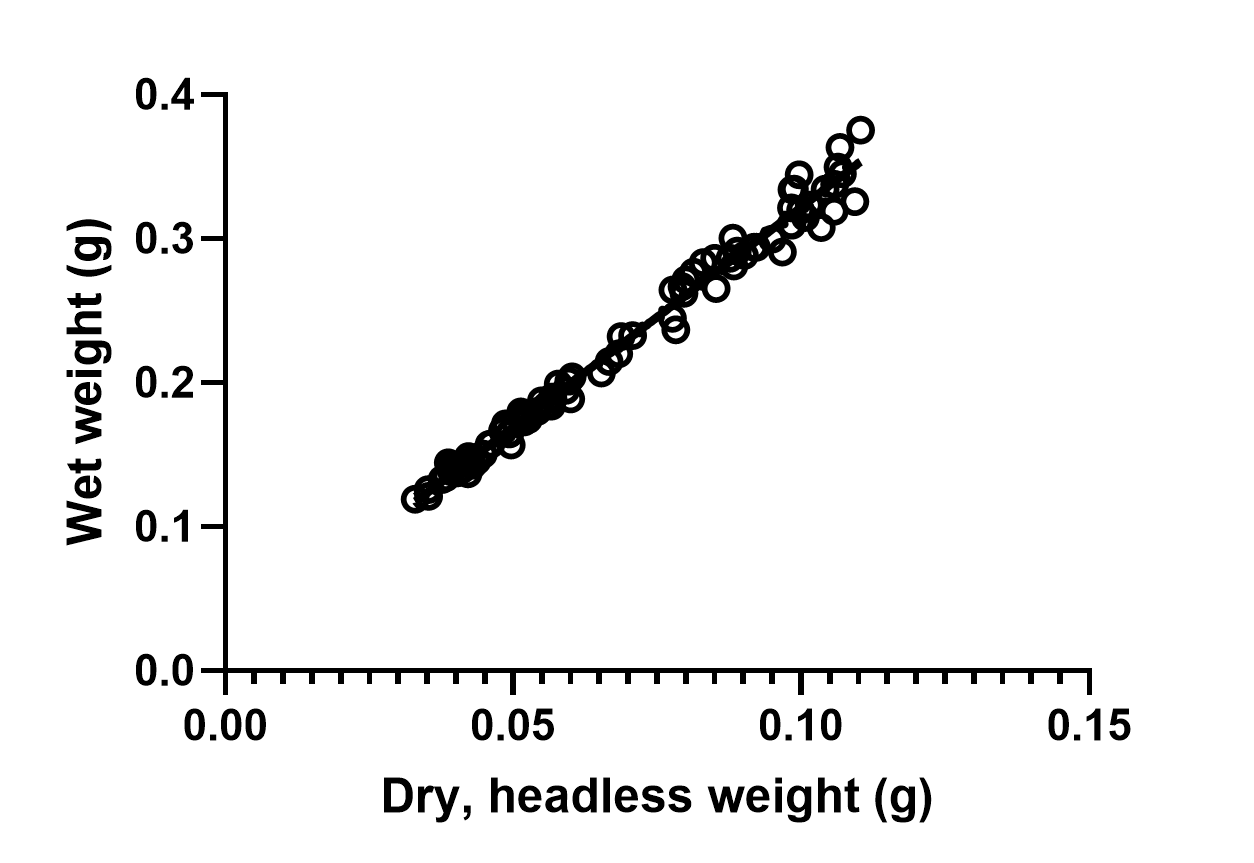
**

**Supplementary Figure 1. Relationship between headless dry weight and wet weight for male *C. pallida.*** [wet weight] = 3.06 [headless dry weight] + 0.02 (F = 5645, df = 85, R^2^ = 0.99, p < 0.0001).

**Supplementary Table 1. Statistics computed with the outlier point included in the analyses.** A bold value in the test column means that a different test was run with the outlier included due to differences in normality. Statistical significance never changed based on the inclusion of the outlier.

| Comparison | Test | Statistics |
| --- | --- | --- |
| Body mass – LM vs. SM  Head width – LM vs. SM | Mann-Whitney t-test  Unpaired t-test | U = 0, p < 0.0001  t = 11.08, df = 16, p < 0.0001 |
| Total brain mass – LM vs. SM  Relative brain mass – LM vs. SM | Unpaired t-test  Unpaired t-test | t = 4.10, df = 17, p = 0.0007  t = 14.75, df = 17, p < 0.0001 |
| Body mass vs. Rel brain mass | Nonlinear regression | y = 2.89 e^-007^ x^2^ - 0.0002 x + 0.05 ; R^2^ = 0.96, df = 16 [comparison of fits to straight line: F = 11.50, p = 0.0037 |
| Log(body mass) vs. log(brain mass) | Linear regression | log(total brain mass) = 0.20 [log(body mass)] + 0.11, F = 27.27, R^2^ = 0.62, p < 0.0001 |
| Total OL mass – LM vs. SM  Total CB mass – LM vs. SM | Brown-Forsythe ANOVA; Dunnett’s T3 | ANOVA: F = 397, p < 0.0001  OL: t = 3.02, df = 15.22, p = 0.017  CB: t = 5.97, df = 16.88, p < 0.0001 |
| Relative OL:CB mass – LM vs. SM | Unpaired t-test | t = 5.29, df = 17, p < 0.0001 |
| Log(body mass) vs. log(OL mass) | Linear regression | log(OL mass) = 0.15 [log(body mass)] + 0.04, F = 14.04, R^2^ = 0.45, p = 0.0016 |
| Log(body mass) vs. log(CB mass) | Linear regression | log(CB mass) = 0.29 [log(body mass)] – 0.61, F = 73.55, R^2^ = 0.81, p < 0.0001 |
| Total cell number – LM vs. SM  Rel OL:CB cell number – LM vs. SM | Unpaired t-test  Unpaired t-test | t = 4.97, df = 17, p = 0.0001  t = 4.97, df = 17, p = 0.0001 |
| OL cell number – LM vs. SM  CB cell number – LM vs. SM | Brown-Forsythe ANOVA; Dunnett’s T3 | ANOVA: F = 204.6, p < 0.0001  OL: t = 5.43, df = 15.01, p = 0.0001  CB: t = 0.18, df = 15.13, p = 0.98 |
| Log(brain mass) vs. log(OL cell number) | Linear regression | log(OL cell #) = 1.64 [log(brain mass)] + 4.91, F = 14.80, R^2^ = 0.47, p = 0.0013 |
| Log(brain mass) vs. log(CB cell number) | Linear regression | F = 0.17, R^2^ = 0.01, p = 0.69 |
